# Supplementary figures and images for: SALARECON connects the Atlantic salmon genome to growth and feed efficiency
Source: PLoS Comput Biol. 2022 Jun 10;18(6):e1010194. doi: 10.1371/journal.pcbi.1010194 (PMC9223387; doi:10.1371/journal.pcbi.1010194)

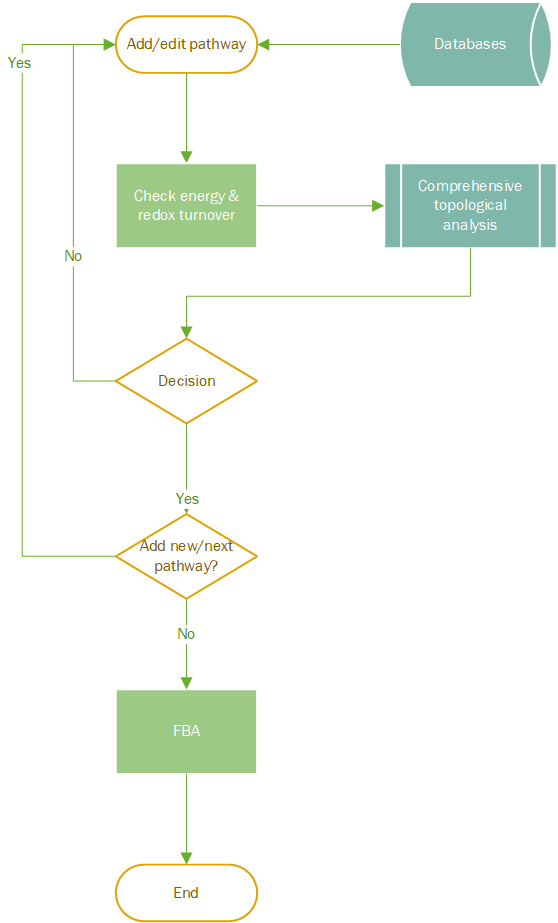

Supplement: S1 Fig — Flowchart showing the procedure used to add new pathways to the draft model or edit pathways already in the draft model. Pathways were added or edited one by one with information about reactions obtained from databases and literature. After adding or editing a pathway, the energy and redox balances and topological properties of the model, e.g. flux consistency, were checked. Based on the results from these analyses, the pathway was either kept or modified. Before final acceptance of a pathway, FBA was performed to ensure that the model was able to predict growth and metabolic fluxes. (TIFF) [file pcbi.1010194.s001.tiff]

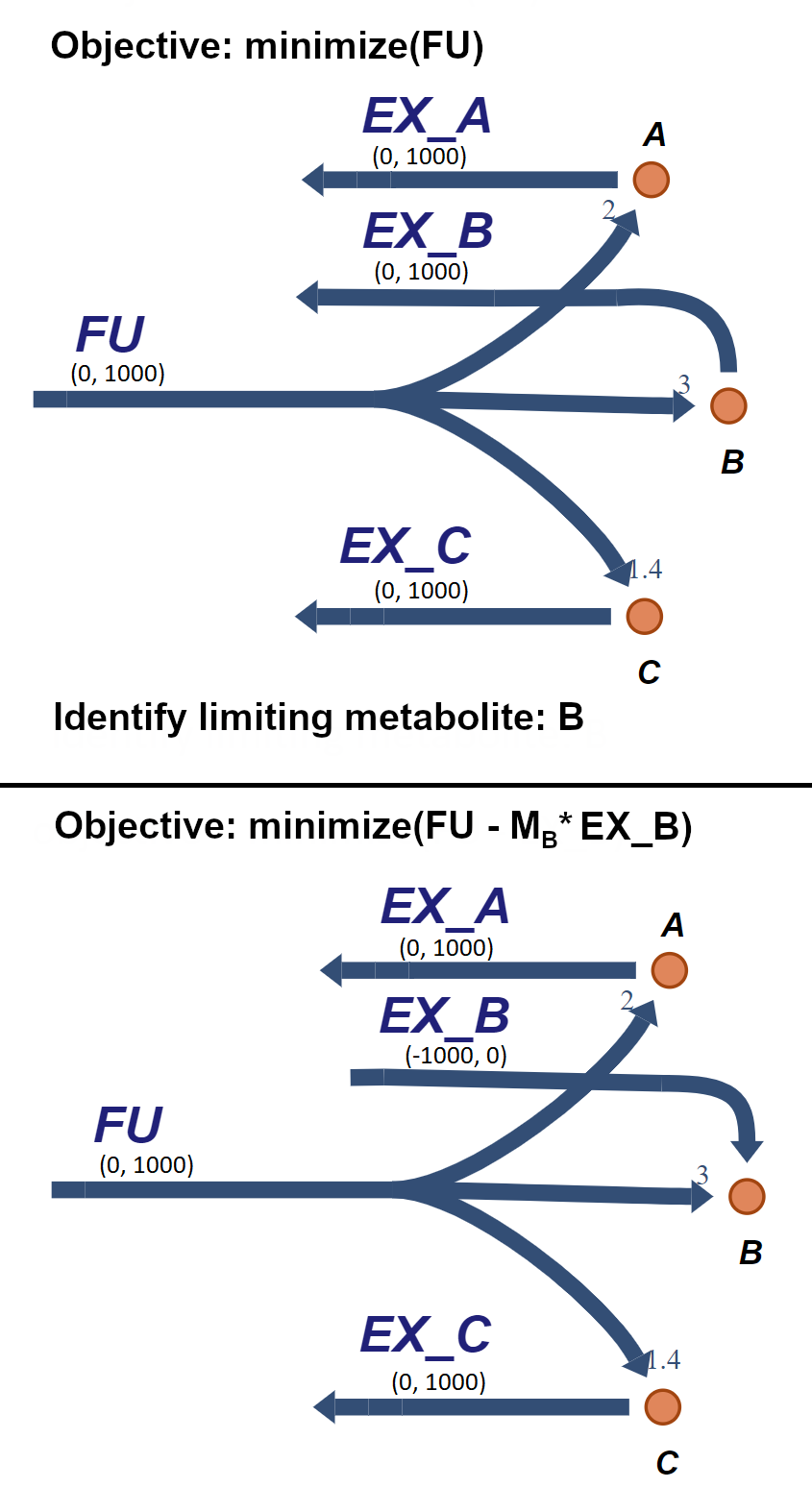

Supplement: S2 Fig — Feed uptake reactions are similar to biomass reactions, but supply metabolites rather than consuming them. The ratios between feed components are represented stoichiometrically, and scaled to sum to 1 g feed per mol uptake, so that one gram of the feed in the figure is equivalent to 2 mol A, 3 mol B and 1.4 mol C. With a fixed growth rate, the minimization of feed uptake is used as the objective of FBA. Surplus of metabolites in the feed uptake reactions are allowed to be exported via exchange reactions to avoid blocking the feed uptake reaction. Limiting metabolites can be identified from the reduced costs of the FBA solution. To avoid large molecules being favored, the reduced cost should be multiplied by the molecular mass (M) of the metabolite. Other factors such as price, CO2 equivalents, or environmental cost could be taken into account in this step. The boundaries of the limiting exchange reaction are reversed to allow uptake, and the reaction is scaled by molecular mass and added to the objective. In this case, the cost of supplements is assumed to be equivalent to mass, but the cost could also be set to be higher than the other feed ingredients, which could be more realistic. (TIFF) [file pcbi.1010194.s002.tiff]

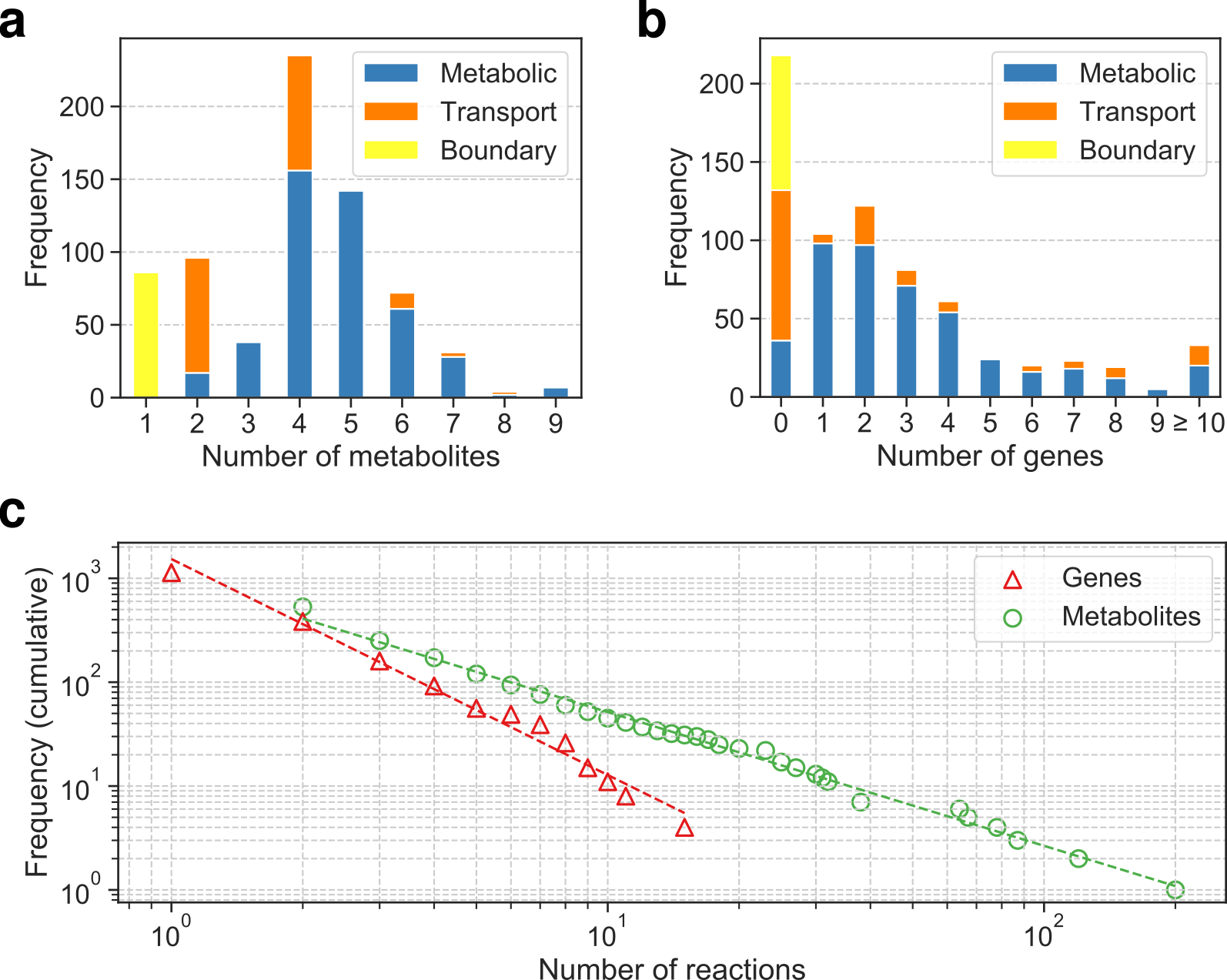

Supplement: S3 Fig — (a) Distribution of number of metabolites converted by reactions. Boundary reactions exchange one metabolite with the extracellular environment and transport reactions usually exchange an even number of metabolites between compartments. (b) Distribution of number of genes associated with reactions. Transport and boundary reactions lack annotation and are not associated with any genes. Most metabolic reactions (95%) are associated with one or more genes. (c) Cumulative distribution of number of reactions associated with genes and metabolites (number of genes or metabolites associated with k or more reactions for all k). Most genes and metabolites are associated with a few reactions but some metabolites are highly connected hubs. Power law fits are shown for genes and metabolites. (TIFF) [file pcbi.1010194.s003.tiff]

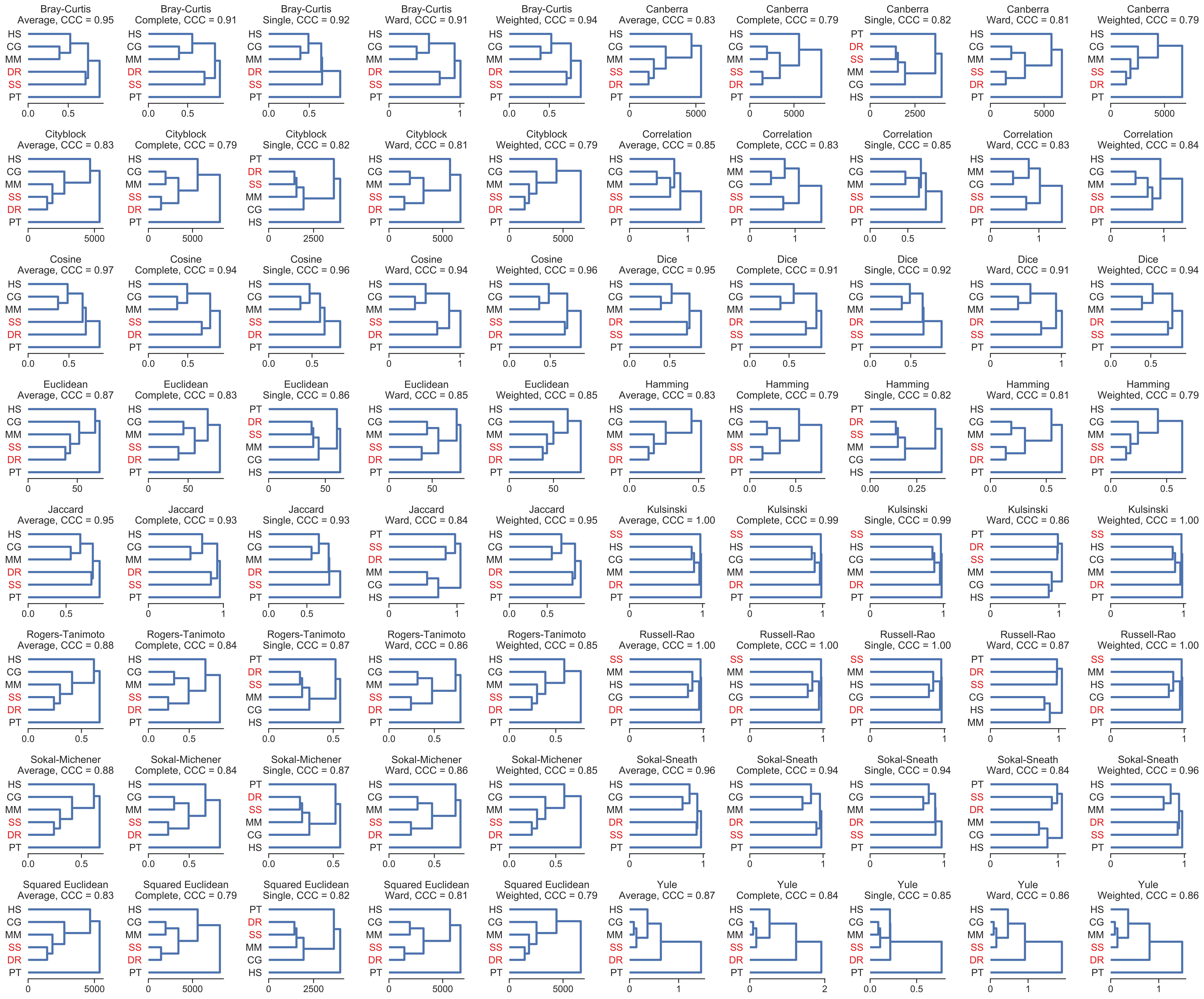

Supplement: S4 Fig — Dendrograms from agglomerative hierarchical clustering of reaction contents of metabolic models of Salmo salar (SS), Danio rerio (DR) [17], Mus musculus (MM) [29], Cricetulus griseus (CG) [30], Homo sapiens (HS) [26], and Phaeodactylum tricornutum (PT) [31]. We combined 16 different dissimilarity measures with five different clustering methods and computed the cophenetic correlation coefficient (CCC) [33] for each measure and method. SS and DR are highlighted in red. (TIFF) [file pcbi.1010194.s004.tiff]

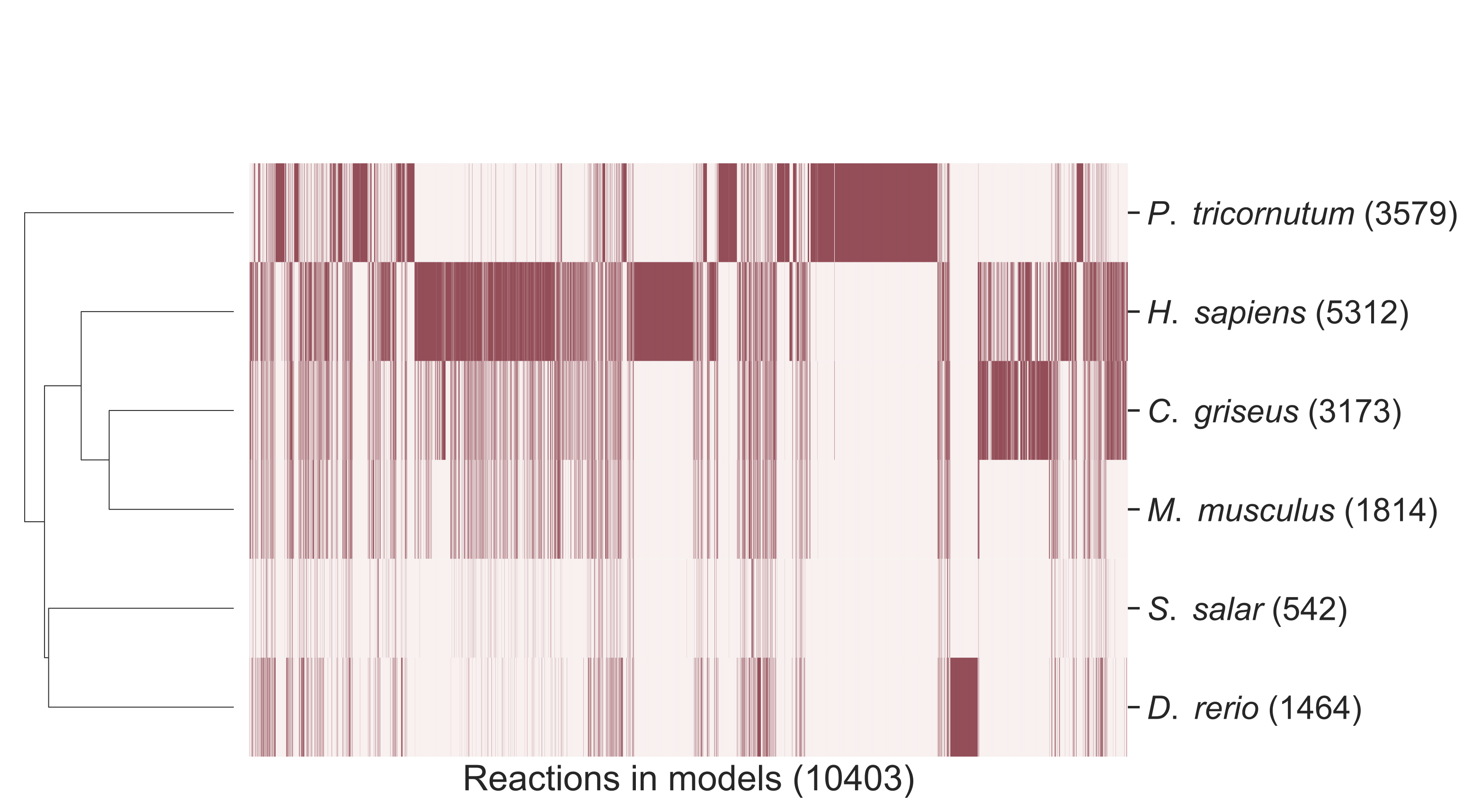

Supplement: S5 Fig — Reaction contents of metabolic models of Salmo salar, Danio rerio [17], Mus musculus [29], Cricetulus griseus [30], Homo sapiens [26], and Phaeodactylum tricornutum [31]. Each row is an organism, each column is a reaction, and a dark cell indicates a reaction that is found in the model of that organism. Rows are clustered by Jaccard distance using the “average” method and the number of reactions is given for each organism. (TIFF) [file pcbi.1010194.s005.tiff]

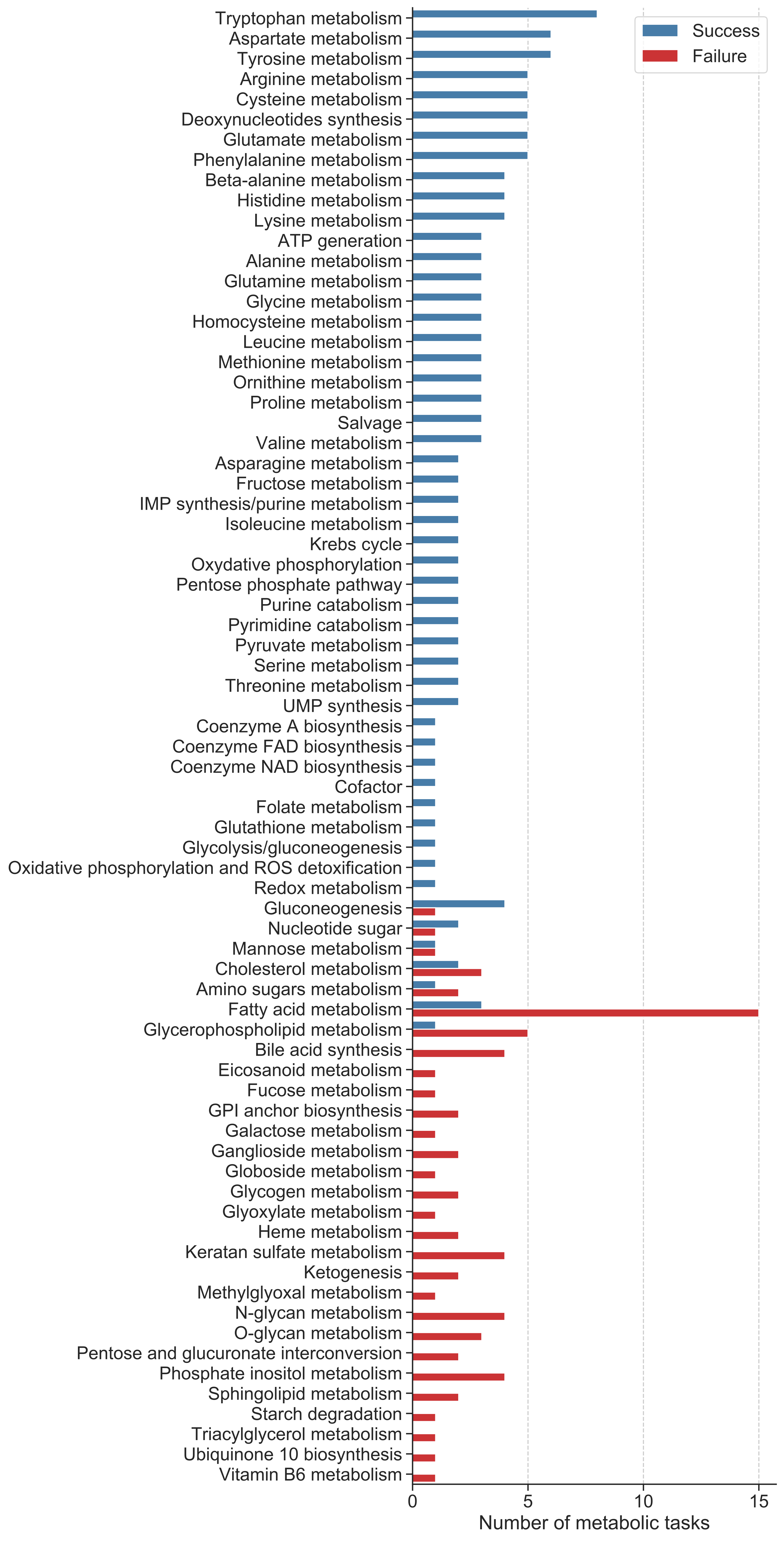

Supplement: S6 Fig — Ability of SALARECON to perform metabolic tasks [35]. Tasks are grouped by metabolic subsystem and classified as successful if model predictions reflected expected metabolic (in)capabilities. (TIFF) [file pcbi.1010194.s006.tiff]

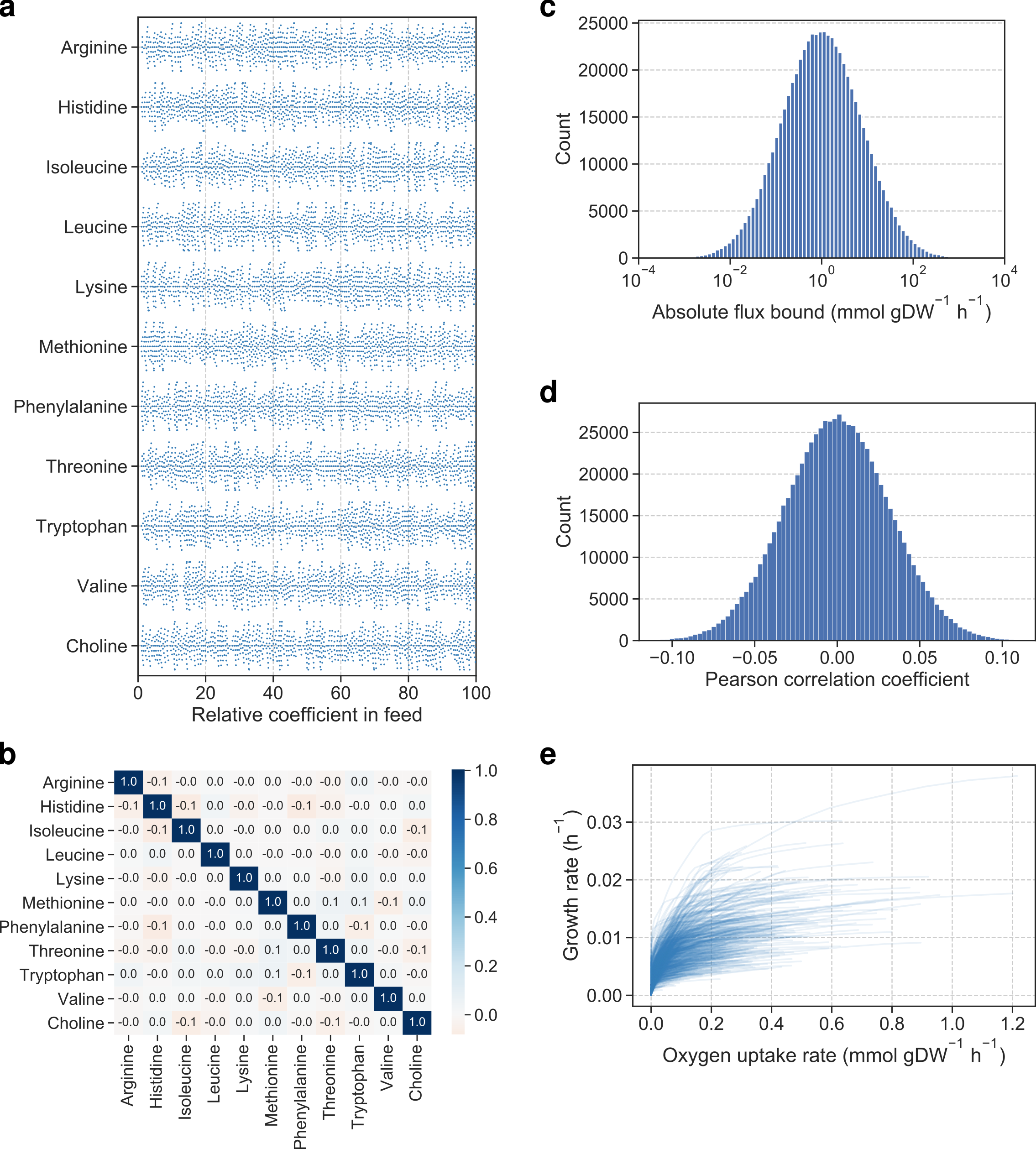

Supplement: S7 Fig — (a) Feed coefficients of amino acids and choline in conditions used to predict oxygen-limited growth (1,000 samples). The coefficients were randomly sampled from a uniform distribution. (b) Pairwise Pearson correlations between metabolites of feed coefficents shown in a. (c) Flux bounds for conditions used to predict oxygen-limited growth (1,000 samples). Flux bounds were randomly sampled from a lognormal distribution. (d) Pairwise Pearson correlations of flux bounds shown in a. (e) Predicted absolute growth rates as a function of absolute oxygen uptake rates for the 1,000 randomly sampled conditions. The absolute growth rates were not intended to be realistic and only relative growth rates were used in the analysis (normalized by maximum growth rate without oxygen limitation). (TIFF) [file pcbi.1010194.s007.tiff]

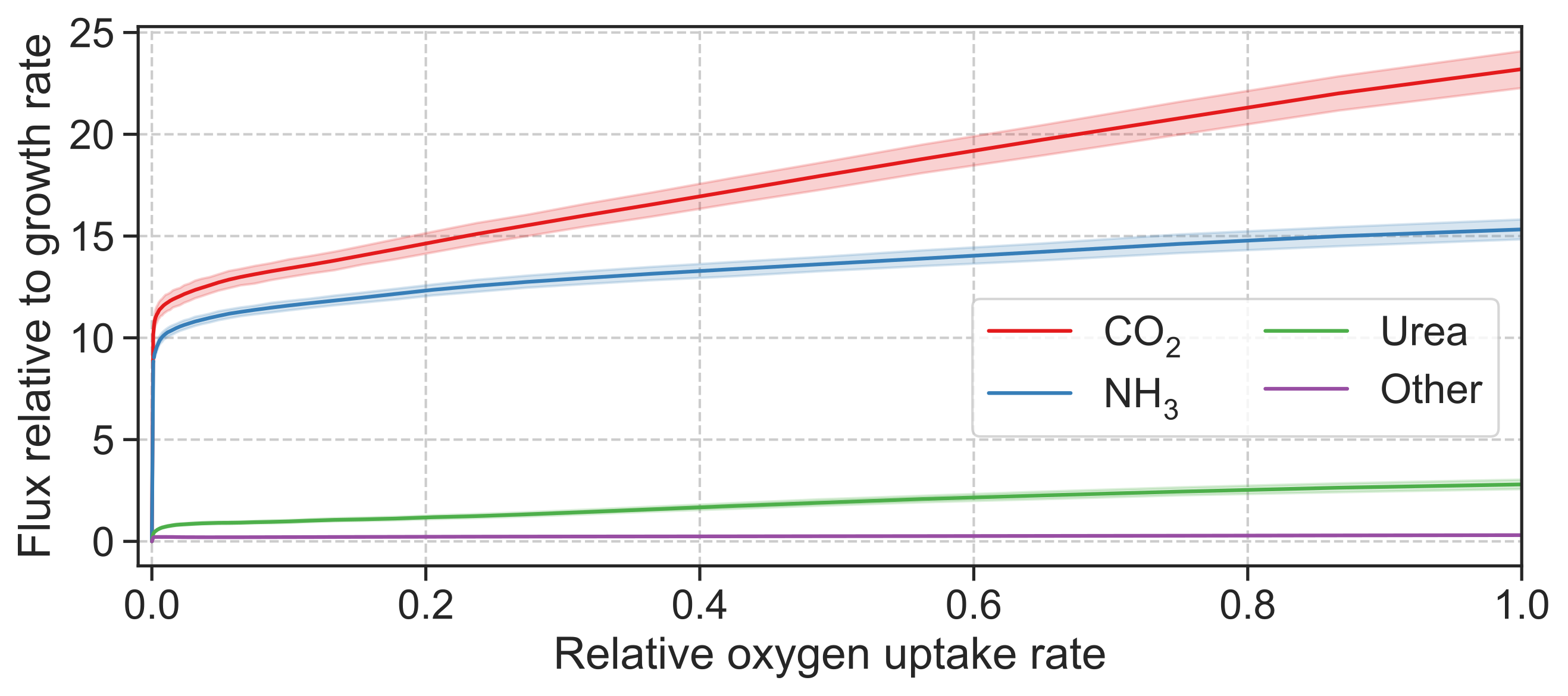

Supplement: S8 Fig — Secretion flux relative to growth rate from oxygen-limited growth simulations. Fluxes are shown for CO2, NH3, urea, and all other secreted metabolites combined. Mean relative flux across 1,000 randomly sampled conditions is shown with 95% confidence bands from bootstrapping with 1,000 samples. (TIFF) [file pcbi.1010194.s008.tiff]

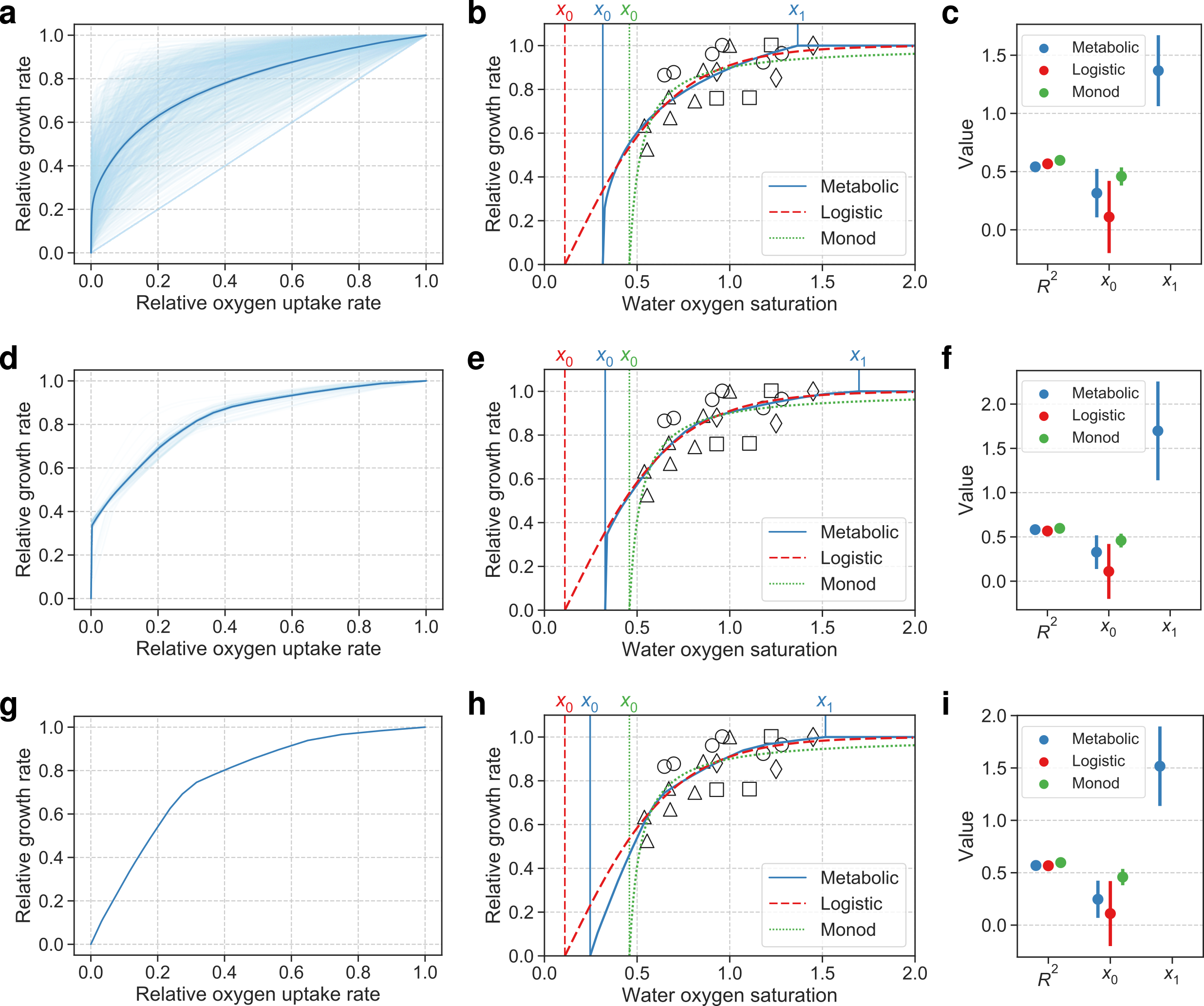

Supplement: S9 Fig — Results from oxygen-limited growth analysis with (a–c) 1,000 randomly sampled feeds and flux bounds, (d–f) 100 randomly sampled feeds with default flux bounds, and (g–i) a fish meal feed (Table 1) with default flux bounds. See legend for Fig 4a–4c. (TIFF) [file pcbi.1010194.s009.tiff]

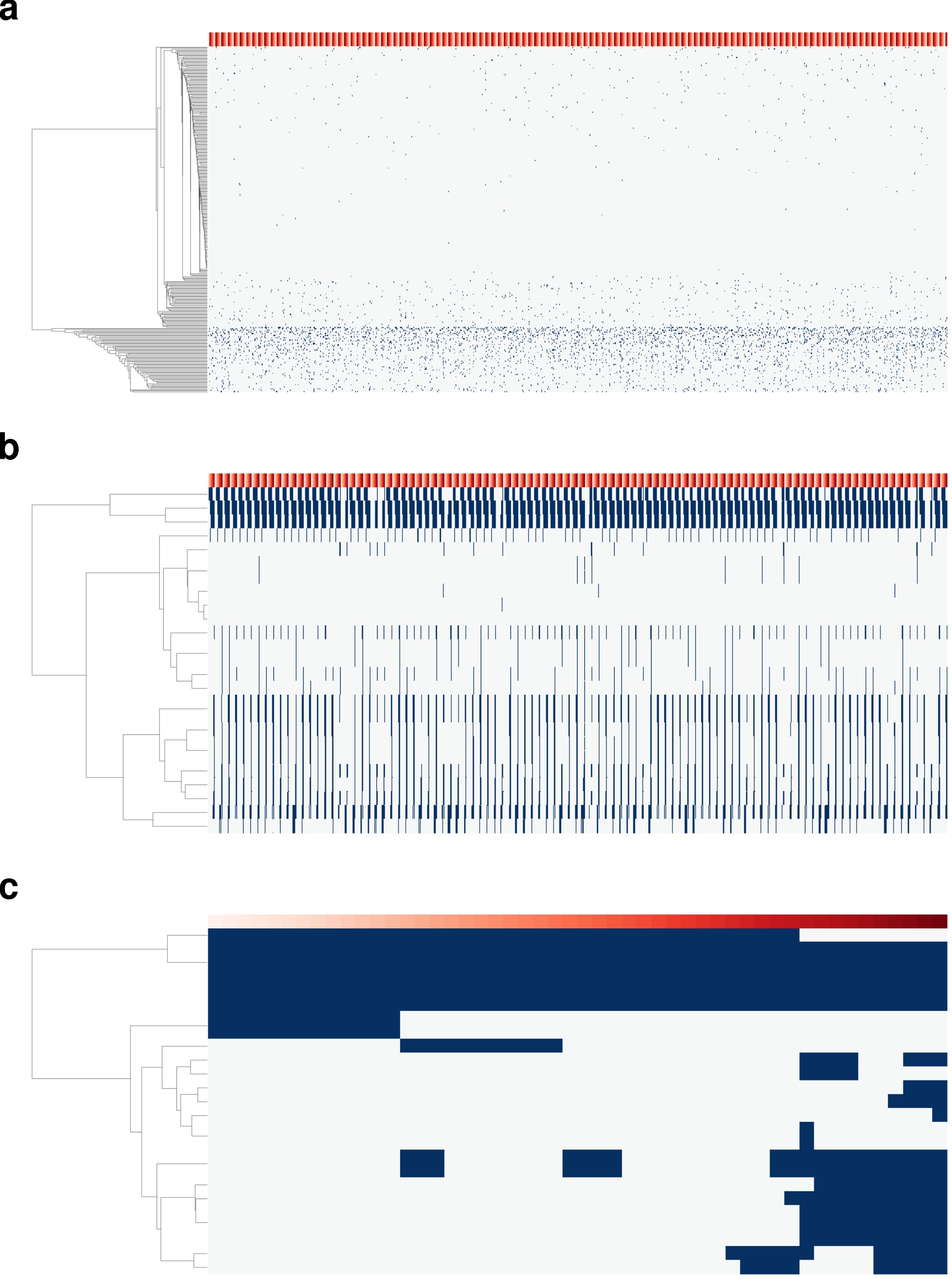

Supplement: S10 Fig — Limiting reactions in oxygen-limited growth analysis with (a) 1,000 randomly sampled feeds and flux bounds, (b) 100 randomly sampled feeds with default flux bounds, and (c) fish meal feed (Table 1) with default flux bounds. Rows are reactions, columns are flux distributions sorted by condition and relative oxygen uptake rate, and a dark cell indicates that a reaction is limiting in a solution (i.e. has flux equal to a non-zero flux bound). Rows are clustered by Euclidean distance using Ward’s minimum variance method. (TIFF) [file pcbi.1010194.s010.tiff]

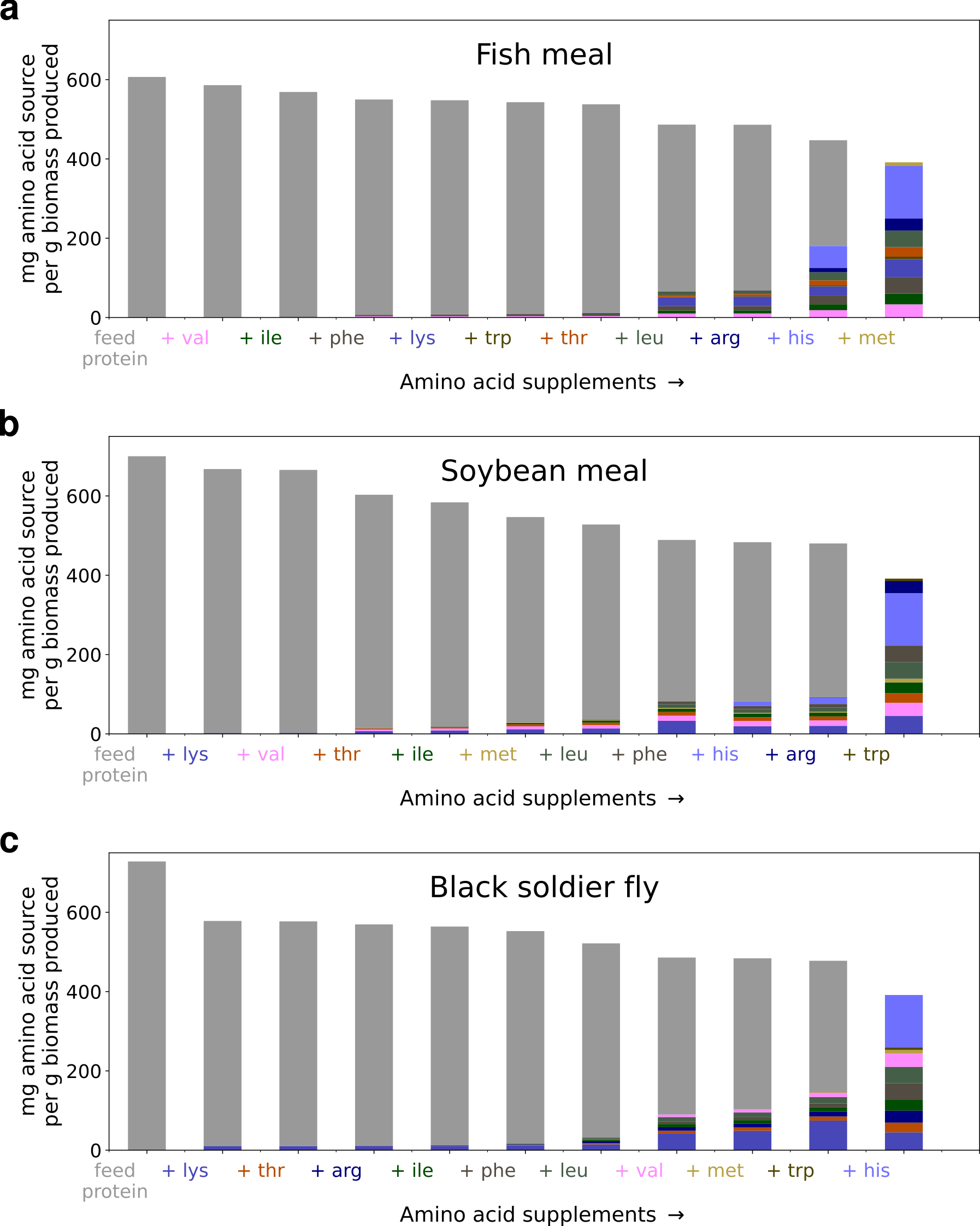

Supplement: S11 Fig — Feed efficiency as a function of number of supplemented amino acids, measured in mg feed ingredient and supplemented amino acids consumed / gDW biomass produced for (a) fish meal, (b) soybean meal, and (c) black soldier fly larvae meal. Amino acids are indicated by color and ordered from most limiting (left) to least limiting (right). Each bar represents the fed amount of amino acid sources, with one amino acid supplemented per step towards the right. Limiting amino acids were supplemented until all feed protein had been replaced. (TIFF) [file pcbi.1010194.s011.tiff]
