# Supplementary material for: SALARECON connects the Atlantic salmon genome to growth and feed efficiency
Source: PLoS Comput Biol. 2022 Jun 10;18(6):e1010194. doi: 10.1371/journal.pcbi.1010194 (PMC9223387; doi:10.1371/journal.pcbi.1010194)
Supplement: Attachment — Submitted filename: salarecon_revision_response_reviewers.pdf [file pcbi.1010194.s012.pdf]

Response to reviewers of PCOMPBIOL-D-21-01635, "SALARECON connects the Atlantic salmon genome to growth and feed efficiency"

We thank the reviewers for their insightful comments and critical assessment of the readability and logic of our paper. Below we offer a point-by-point response to their remarks, in most cases amending the text to either clarify or refine our analysis and arguments.

Reviewer #1

Salmon is indeed a commercially very valuable fish in the market and it has high global demand. Therefore, in order to meet the global demand, it is produced at a higher scale. However, the challenge is to get the feed correct (similar to the level of marine feed) so that good fish quality is maintained. In this work, the authors have developed the genome-scale metabolic model of salmon fish and then used the model as a tool to examine a different type of commercial fish feed. The model predictions with regards to different feed and supplementation of few amino acids are in line with other experimental observations. Furthermore, the model is capable of mimicking hypoxic growth. Overall, the procedures followed to obtain the model are standard. I believe that the model will be used as a valuable tool to study salmon metabolism and growth.

In my view, the manuscript (the main text) is written very well but the quality of the figures is very poor. In most of the figures, I cannot read what is written in x and y-axis labels. Other texts on the figures are unclear, therefore, I am afraid, it is difficult to judge the quality of the paper. I am happy to go through the paper once the good-quality figures are provided.

Having checked the figures in the PDF generated by the manuscript submission system, we note that every figure includes a link to a high-resolution version (see screenshot below). We hope that this provides a good basis for the reviewers to assess our figures.

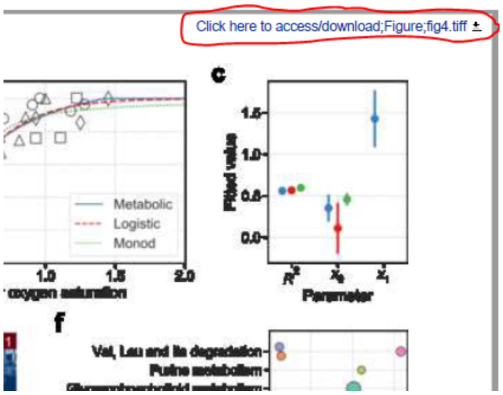

Other than figures (results), which need additional review, I have few more comments.

Line 47-50: Authors mention that due to lack of information, GPR is converted to OR type for all reactions. Is it normal to do so? I believe such detailed experimental information is not available in zebrafish as well as other higher organisms, so how do those models keep GPR relations?

The original choice of OR relationships reflects the Ss4R gene duplication in the ancestral salmonid (Lien et al. 2016), based on the null hypothesis that duplicates retain their function. However, we have improved the GPR relationships of the model using sequence homology between salmon and human and the GPR relationships in the latest human model. This increased the number of genes in the model slightly from 1104 to 1133 and led to GPR relationships for 399 of the 494 enzymatic reactions (81%).

We have updated Figs. 2 and S1 to reflect the increased number of genes as well as our description of the model construction under Methods, which now includes the following: "To infer gene-protein-reaction (GPR) associations for reactions, we mapped Atlantic salmon genes to human homologs and copied GPR associations from the most recent human model."

Analysis of oxygen-limited growth is not clear to me. I did not understand what is 100 randomized conditions? Is it 100 randomly chosen nutrients from the list of all exchange metabolites? Or do you have a media setup and then you chose the reaction bound randomly 100 times? In minimal feed, authors mention amino acids and choline. Which amino acids are essential here?

Each condition is defined by randomly sampled ratios of nutrients and randomly sampled upper bounds for reactions. We have significantly revised the oxygen-limited growth analysis and expanded and clarified its description under Methods, which now includes the following explanations:

"We used parsimonious FBA (pFBA) to find maximal growth rates and minimal flux distributions for 1,000 randomized conditions and 50 logarithmically spaced oxygen uptake rates. (...) For each condition, we uniformly sampled random ratios (1-100) of nutrients in a minimal feed (essential amino acids and choline) that were used as coefficients in a boundary reaction representing feed uptake. (...) We did not allow uptake of any other compounds than essential amino acids, choline, phosphate, and oxygen under any condition. (...) To account for uncertainty in relative flux capacities and ensure that no single reaction was always growth-limiting, we also sampled random bounds for all reactions for each condition."

Line 117: Authors minimize the feed uptake. In my opinion, it needs a more detailed explanation. Is it one of the objective function reactions where all feed constituents are lumped into a feed reaction, similar to one in the biomass? Or do you minimize uptake reactions one by one iteratively while keeping the rest constant? I did not fully understand this part.

We have added a supplementary Fig. S2 with a legend that explains this analysis as follows:

“Feed uptake reactions are similar to biomass reactions, but supply metabolites rather than consuming them. The ratios between feed components are represented stoichiometrically, and scaled to sum to 1 g feed per mol uptake, so that one gram of the feed in the figure is equivalent to 2 mol A, 3 mol B and 1.4 mol C. With a fixed growth rate, the minimization of feed uptake is used as the objective of FBA. Surplus of metabolites in the feed uptake reactions are allowed to be exported via exchange reactions to avoid blocking the feed uptake reaction. Limiting metabolites can be identified from the reduced costs of the FBA solution. To avoid large molecules being favored, the reduced cost should be multiplied by the molecular mass (M) of the metabolite. Other factors such as price, CO_2 equivalents, or environmental cost could be taken into account in this step. The boundaries of the limiting exchange reaction are reversed to allow uptake, and the reaction is scaled by molecular mass and added to the objective. In this case, the cost of supplements is assumed to be equivalent to mass, but the cost could also be set to be higher than the other feed ingredients, which could be more realistic.”

Line 129: I am surprised to see this low percentage of metabolic gene coverage in the model. Only 48% of kegg metabolic genes are covered in the model. Why are the rest 52% of metabolic genes from kegg not included? I think this is a very big percent of genes to ignore -- and they are mostly metabolic genes as given in the KEGG. I am not saying all kegg metabolic genes should be included but leaving 52 % is too much I think. I think this low coverage of metabolic genes is the main reason why the model achieved only 53% MEMOTE score. If the aim is to understand the specific tasks so, in that case, can we not say that the reconstructed model is a core model considering only central pathways/reactions?

The reviewer is correct that the model focuses on core metabolism, specifically energy, amino acid, and nucleotide metabolism. This issue was raised by all reviewers and we have now rewritten the manuscript to make the scope of the model clear from the very beginning (Abstract and Introduction) as well as throughout the text (Methods, Results, and Discussion). Specifically, we have made the following clarifications:

Abstract: "We present SALARECON, a model focusing on energy, amino acid, and nucleotide metabolism that links the Atlantic salmon genome to metabolic fluxes and growth.”

Introduction: “Here, we present SALARECON: a metabolic model built from the Atlantic salmon genome that predicts growth and metabolic fluxes. It has been manually curated to ensure flux consistency and focuses on energy, amino acid, and nucleotide metabolism.”

Methods: “We manually built a draft model focusing on Atlantic salmon energy, amino acid, and nucleotide metabolism using the genome with annotations from KEGG and the software Insilico Discovery.

Results: “The model focuses on energy, amino acid, and nucleotide metabolism and covers 1,133 genes, which amounts to 2% of the 47,329 annotated genes in the genome and 50% of the 2,281 Atlantic salmon genes that are associated with metabolic reactions in KEGG. (...) SALARECON correctly captured all expected metabolic (in)capabilities for the three metabolic systems within the scope of the model (energy, amino acid, and nucleotide metabolism).”

Discussion: "Still, SALARECON performs very well according to all of our metrics: it (...) and performs all metabolic tasks within the scope of the model (amino acid, nucleotide, and energy metabolism)."

The fact that about half of the metabolic genes in KEGG are included in the model is consistent with its scope. However, the MEMOTE gene annotation score of 53% was due to something else, namely the list of databases that are included in MEMOTE and checked for annotation. We have added refseq, ncbigi and ncbiprotein in response to the reviewer's concern, and now get a gene annotation score of 71% in MEMOTE. Atlantic salmon genes are not included in all of the databases checked by MEMOTE, and we believe that the currently included mappings are sufficient for all users.

Reviewer #2

The authors have 1) reconstructed a metabolic model of Atlantic salmon, 2) conducted a number of tests of the model both standardized and custom, the later including its Jaccard distance to other models; essentiality of amino acids; relative growth rate with randomly sampled nutrient uptake and flux capacities, 3) used the model to evaluate the effect of amino acid supplements to different feeds. While new metabolic models are always useful and needed, the paper seems too limited in biological insight and in motivation of its methodology, as detailed in the following specific comments.

We thank the reviewer for the very constructive criticism of our work. Our resubmitted manuscript has been substantially revised, particularly to demonstrate the biological relevance of the model and to clarify the description and motivation of methods.

Major comments

The authors interpret the Jaccard distance as phylogenic distance. However, other factors may also influence the metric, e.g. the size or focus of the reconstruction, in figure S2 it is reported that the different models span a range of 456-3554 reactions.

The reviewer points out important aspects of the model comparison. We do not claim that the Jaccard distance can be directly interpreted as a phylogenetic distance. Rather, we observe that the Jaccard distance between models based on presence and absence of reactions produces a tree that qualitatively recapitulates known phylogenetic relationships.

As discussed by others, "the Jaccard distance is the de facto standard to measure similarity between genome-scale models" [1]. Importantly, it has been shown that Jaccard distance based on presence and absence of reactions can reflect known phylogeny and phenotypic differences [2]. We therefore argue that this metric is appropriate for comparing our model to other models.

We have added this sentence to Methods with a reference to [1]: "We used Jaccard distance because it is the most common metric for measuring the similarity of metabolic models".

1. *Cabbia et al. A Distance-Based Framework for the Characterization of Metabolic Heterogeneity in Large Sets of Genome-Scale Metabolic Models. Patterns 1.6 (2020).* https://doi.org/10.1016/j.patter.2020.100080
2. *Magnúsdóttir et al. Generation of genome-scale metabolic reconstructions for 773 members of the human gut microbiota. Nature Biotechnology 35.1 (2017).* https://doi.org/10.1038/nbt.3703

The model constitutes a unique opportunity to compare Salmon metabolism to that of other organisms, but no fish specific biological conclusions seem to be drawn, e.g. differences in nitrogen excretion or oxygen utilization. It is not clear how the results would differ if a model from another organism was used, assuming that the biomass equation was amended. Also differences in arginine metabolism between fish and mammals would be interesting to discuss, arginine is only considered a conditionally essential amino acid in human. The reference cited for its essentiality in salmon does not seem to be the original source of the claim.

We appreciate this point and have added an analysis comparing SALARECON to the latest human model to show that SALARECON enables simulation of fish-specific metabolism. Specifically, we found minimal sets of metabolite uptakes and secretions that allow growth for human and salmon and found that SALARECON differs from the human model not only in essential amino acids (Arg is essential for salmon) but also allows simulation of growth with carbon dioxide and ammonia as the only waste products in agreement with basic fish physiology. The human model required additional waste products and could dispose of nitrogen equally well through ammonia, urea, or urate, although urea is the major nitrogenous waste product in human and other mammals.

We have expanded Fig. 3 and substantially revised the text to accommodate this new analysis:

Methods: "Finally, we evaluated the ability of SALARECON to capture fish-specific metabolism by comparing metabolite uptake and secretion to the most recent human model. Specifically, we enumerated minimal growth-supporting uptake and secretion sets for both models using scalable metabolic pathway analysis. We required non-zero growth rate, uptake of oxygen and essential amino acids, and secretion of carbon dioxide as well as ammonia, urea, or urate. We enumerated minimal uptake sets first and then allowed uptake of all metabolites found in uptake sets before enumerating minimal secretion sets."

Results: "Finally, to test the ability of SALARECON to capture basic fish physiology, we compared it to the latest human model, RECON3D, by computing minimal sets of metabolite uptakes and secretions that allow growth. In addition to oxygen and essential amino acids, SALARECON required uptake of choline, a lipid precursor, and phosphate, an essential nutrient for fish that is supplemented in salmon feeds. The only secretions needed to support growth were carbon dioxide and ammonia. Notably, we found that secretion of urea was also possible, but not sufficient to support growth without secretion of ammonia. In line with this, Ammonia is the major nitrogenous waste product in fish with urea a comparatively minor contributor. RECON3D is much larger than SALARECON and therefore allowed for a wider range of lipid precursors (27 options). It also required secretion of a carboxylic acid (11 options) and a lipid byproduct (132 options) in addition to carbon dioxide and a nitrogenous waste product. Urea is the major nitrogenous waste in mammals, but RECON3D could grow while secreting ammonia or urate as well."

We have also added a detailed analysis of secretion fluxes to our oxygen-limited growth analysis. Here, we found that carbon dioxide and ammonia are by far the most important secretion products across all oxygen levels and 1000 randomly sampled conditions. The third most important secretion product was urea, but with a much smaller secretion flux than ammonia. All other secretion products combined made a very small contribution to total secretion flux. The fact that the model "chooses" secretion of carbon dioxide and ammonia across pFBA solutions for thousands of random conditions supports our claim that SALARECON captures fish-specific metabolism.

We have added a new Fig. S7 showing secretion fluxes from the oxygen-limited growth analysis and added the following text to Results: "Supporting our claim that SALARECON captures fish-specific metabolism, we found that the major secretion products across all oxygen levels and sampled conditions were CO_2 and NH_3. Urea had the third highest secretion flux but this was much smaller than the secretion flux for NH_3, and the secretion fluxes of all other secreted metabolites combined was vanishingly small."

Regarding amino acid (and particularly Arg) essentiality, we have added an additional reference supporting Arg as essential in salmon and other fish: Andersen, S. M., Waagbø, R. & Espe, M. Functional amino acids in fish health and welfare. Front. Biosci. ***1****, 143–169 (2016).*

The method to randomly sample input and flux constraints appears to be novel since no references are given, but it is not thoroughly motivated, described, or analyzed, e.g. what would the effect be of only sampling inputs or constraints? Is it reasonable to assume that only essential amino acids are provided and that they are uniformly distributed? How is the sampling of constraints motivated? How is this approach expected to fair with respect to well-known biases from sampling of highly interdependent fluxes (Heinonen 2019). The results of this method would require careful study, to rule out unintended effects of the imposed constraints, e.g. in the analysis of the flux distributions the flux through glycolysis is implicitly assumed to be energy-generating, however, since uptake of glucose is blocked, and glycogen is in the biomass equation, the flux is likely reversed (gluconeogenesis). The manuscript mentions blockage of nutrient secretion of feed nutrients, does this mean that the constraint only applies to the amino acids, what would be the consequence of not doing so?

We have significantly revised the oxygen-limited growth analysis and expanded and clarified its description under Methods, as also mentioned in our reply to reviewer #1. This includes a revised random sampling approach with references supporting our choices. For the sampling of nutrient ratios in the feed, we do not have prior information and therefore choose to sample from a uniform distribution. For the sampling of upper bounds for reactions, we have now replaced the uniform sampling by lognormal sampling with justification and references for this given in the text below. It is important to note that we sample bounds for fluxes rather than the fluxes themselves, so the caveats that would apply to random flux sampling do not apply here.

Reviewer #2 raises a good point about gluconeogenesis, but we would argue that it is not unreasonable to assume that there will often be net flux towards glucose in salmon. There is hardly any sugar or carbohydrates in the natural or fed diets of salmon, meaning that glucose

will have to be synthesized from other nutrients obtained from the diet. The activity of glycolysis/gluconeogenesis likely varies between tissues and activities as well, which is not currently captured by the model.

In addition to the changes in the text given in our reply to reviewer #1, we have added the following to Methods:

"To account for uncertainty in relative flux capacities and ensure that no single reaction was always growth-limiting, we also sampled random bounds for all reactions for each condition. The upper bound r_{max} of an enzymatic reaction is determined by the turnover number k_{cat} and total enzyme concentration [E]: $r_{max} = k_{cat}$ [E]. Approximately lognormal distributions have been observed for both k_{cat} (Bar-Even et al. 2011) and [E] (Madhukar et al. 2015), and the product of two lognormal random variables is also lognormal. We therefore sampled r_{max} from a lognormal distribution with mean 0 and standard deviation 2 for the natural logarithm of r_{max}, allowing upper flux bounds to vary by approximately six orders of magnitude We kept the original reaction reversibilities and sampled bounds for reversible reactions separately for each direction."

A set of fitting-parameters and relative rates are used to fit the model to experimental data, this, however, seems unnecessary, since the minimum oxygen uptake should follow from the oxygen uptake required for maintenance ATP expenditure and the maximum oxygen uptake should depend on the maximum specific growth rate together with the growth associated ATP expenditure, i.e. more explanatory parameters that could be estimated from the data.

While we agree that this is an important future development, our current data sources do not enable the construction of a realistic non-growth-associated maintenance term in the model. A key point of fitting the model predictions to oxygen-limited growth data was to show that SALARECON can give good estimates of physiological parameters (minimal and maximal water oxygen saturation) and give detailed explanations of the predictions that led to those estimates in terms of metabolic fluxes, none of which would be possible with the simpler growth models.

Fish meal is used as reference in Figure 5, but presumably a similar conclusion could more easily be drawn by comparing the optimal amino acid composition identified by the model to the to the composition in the feed. Since only essential amino acids are considered, the interconversion capacity of the model will likely not be utilized, and the optimal composition is therefore presumably highly similar to the amino acid composition in biomass, in particular since unlimited glucose is supplied so that the possibility for ATP constrained growth is neglected. This, btw, does seem to be a reasonable assumption under optimized growth conditions (El-Mowafi 2010). On a more general level the feed optimization assumes no effects of gut microbiota or obligate losses, which perhaps should be discussed.

Reviewer #2 is correct that the optimal amino acid composition according to the model (biomass composition) is important for this analysis. To make it easier to interpret our results, we have added the amino acid compositions of biomass and the three feed ingredients to Fig. 5. However, we still compare limiting amino acids in soybean and insect meal to fish meal because

fish meal is the standard for comparison of salmon feed ingredients and because this is the comparison for which we have data on differences in amino acid limitation.

We thank the reviewer for the suggested reference, which we have incorporated into our Methods section as follows: "This is supported by evidence that reducing feed amino acid levels has a negative effect on feed intake regardless of dietary energy level (El-Mowafi et al. 2010)."

Reviewer #2 is also correct to point out that the gut microbiota potentially has a significant effect on feed consumption, and this is something that we are working on for a future publication. We also discuss this briefly in Discussion: "We will also leverage automated metabolic reconstruction tools for microbes to build models of the Atlantic salmon gut microbiota. By coupling tissue-specific models to each other and to gut microbiota models, we can make detailed and partially dynamic whole-body models."

Minor comments

There seems to be no reason to use the arbitrary specific growth rate of 1 h-1, which is unphysiologically high. Similar to elsewhere in the manuscript yields, gdw/gdw/mmol O2 could be reported.

We only use relative values, never absolute values, for growth and feed uptake rates in our analyses. We have now edited Methods to clarify this:

"We normalized feed uptake to the same total mass (g gDW^{-1} h^{-1}) to ensure that conditions were comparable, but the absolute value was arbitrary as only relative predictions were needed."

"For each feed ingredient, we deactivated import of amino acids via other boundary reactions, fixed the growth rate to the same value (arbitrary, as we were interested in generated biomass relative to consumed feed), and normalized feed uptake to the same total mass (g gDW^{-1} h^{-1}) before minimizing feed uptake flux."

Figure 4d could be improved for interpretability. How sensitive are the clusters to the 100 random samples, if 1000 samples were used, would the figure look the same?

We have now increased the number of samples to 1000. The results remained similar, although with minor differences from the previous version of the manuscript due to changes in the random sampling of upper flux bounds.

Due to differences in specific activity between enzymes, minimized flux is not the same as minimal enzymatic cost (line 196).

The term "minimal enzymatic cost" was inaccurate and we have removed it from the manuscript.

On line 129 it is stated that the model focuses on energy metabolism, but on line 279 it is stated that it focuses on amino acid, nucleotide, and energy metabolism. This a more accurate description, since most of the results relate to amino acids.

We agree. As shown in detail in our reply to reviewer #1, we have revised the whole manuscript to make this point very clear.

It is unusual to see the Monod model with an intercept term as in figure 4b.

We use a Monod model and a logistic model as examples of simple growth models that we can compare to SALARECON in our analysis of oxygen-limited growth. The Monod model is typically used for microbial growth which typically starts immediately when a substrate becomes available. For oxygen-limited growth in salmon, we know that growth does not start immediately when oxygen saturation becomes non-zero; there is a lower oxygen saturation bound above which growth starts and we added the intercept to the Monod model to allow for this. To be clear, we are not advocating the use of this model in general or to study fish growth in particular.

References

Markus Heinonen, Maria Osmala, Henrik Mannerström, Janne Wallenius, Samuel Kaski, Juho Rousu, Harri Lähdesmäki, Bayesian metabolic flux analysis reveals intracellular flux couplings, Bioinformatics, Volume 35, Issue 14, July 2019, Pages i548–i557

El-Mowafi, A., Ruohonen, K., Hevrøy, E.M. and Espe, M. (2010), Impact of digestible energy levels at three different dietary amino acid levels on growth performance and protein accretion in Atlantic salmon. Aquaculture Research, 41: 373-384.

Reviewer #3

The authors present a metabolic model of Atlantic salmon. They compare the model to genome-scale models of other organisms, and use the model for looking into areas of metabolism that change under varying feed/oxygen uptakes, and detecting specific amino acids that are limiting in different feed sources. Overall the paper is well written and is a good foundation stone for salmon modeling work to come. I commend the authors for using several state of the art practices in model development, such as unit testing and memote validation. However, there are a few major points that should be addressed in the manuscript and model before publication.

Major comments

* L37: The description of how the draft model was generated is extremely brief; a more thorough explanation of the methods used is required, especially considering that a) the most important result of this manuscript is the model itself, and b) the authors used proprietary software for it, which hinders reproducibility. Was a template model used to build this reconstruction, or was it built from scratch? Which alignment method was use to map the genes to KEGG, or was that information directly taken from the annotated genome? Which criteria were used for adding reactions to the draft model? Which criteria were used to finish adding reactions? Was there any gap-filling performed? How did the authors ensure the model could produce biomass?

We have significantly expanded our description of the draft model construction in Methods and added a new Fig. S1 that shows how new pathways were added to the model in a flowchart. We did not use a template model but obtained information about genes, reactions, and metabolites from databases and literature. Salmon genes are already in KEGG so no additional mapping was required. We added reactions to the draft model when there was genetic evidence for those reactions, and we stopped adding reactions to the final model when we were satisfied with the results of our quality evaluation (Fig. 3), specifically that the model could simulate growth with FBA, clustered with zebrafish in terms of reaction contents, performed all metabolic tasks within the model scope, had a MEMOTE score close to 100%, and correctly predicted essential amino acids. We note that all steps in the model construction after draft model construction are fully reproducible with code that is available in our git repository.

The updated text for draft model construction in Methods is as follows:

"We manually built a draft model focusing on Atlantic salmon energy, amino acid, and nucleotide metabolism using the genome \cite{Lien2016} with annotations from KEGG \cite{Kanehisa2019} and the software Insilico Discovery (Insilico Biotechnology, Stuttgart, Germany). Pathways were added or edited one by one with information about reactions obtained from databases and literature. After adding or editing a pathway, the energy and redox balances and topological properties of the model, e.g., flux consistency, were checked. Based on the results from these analyses, the pathway was either kept or modified. Before final acceptance of a pathway, FBA was performed to ensure that the model was able to predict growth and metbabolic fluxes. We used WoLF PSORT with default settings through SAPP to assign metabolites and reactions to six different compartments (cytosol, mitochondrion, inner mitochondrial membrane, extracellular environment, peroxisome, and nucleus). Exchange reactions were added to allow metabolite import (negative flux) and export (positive flux)."

* L47: Assuming every gene-reaction-relationship is an OR relationship is a quite strong assumption, that will lead to many false negatives when using the model for gene essentiality predictions (one of the most common uses for metabolic models). Most reconstruction tools are able to account for complexes; if insilico discovery cannot do it, I would suggest, for instance, using a reference model for retrieving this information: if the same reaction is present in both models, and there is homology between the corresponding genes, the AND/OR relationship from the reference can be copied.

This is similar to a point made by reviewer #1. The choice of an OR relationship reflects the Ss4R gene duplication in the ancestral salmonid (Lien et al. 2016), based on the null hypothesis that duplicates retain their function. That said, we have improved the GPR relationships of the model using sequence homology between salmon and human and the GPR relationships in the latest human model. For further details, see our reply to reviewer #1.

* L78: This section reads quite confusing and it took me a few reads to understand what the authors did. Considering that most of the result section is about the oxygen simulations, the authors should either make this section clearer to read, or remove focus from it to highlight the (much clearer) next section "Predicting growth-limiting amino acids in feeds".

This point was also brought up by reviewer #1. We agree that it needed clarification, and we have expanded and clarified the analysis as summarized in our replies to reviewers #1 and #2.

* L141: It is misleading to claim that this model accounts for the metabolic production of lipids, as the only lipid accounted for in the biomass reaction is PC 16:0/16:0, i.e. most of the rich lipid metabolism that fish has is ignored in the model, and the uptake costs of choline are largely over-estimated. Similarly, carbohydrates are only represented with glycogen. Considering the many published semi-automated tools for model generation, have the authors considered adding some additional metabolic content to their model so that it can produce some additional biomass constituents, e.g. lipids and/or carbohydrates, but also vitamins, cofactors, trace minerals? If not, then at the very least the authors should consider removing mentions to those pathways as being fully accounted for in the model.

Refining lipid metabolism is on our roadmap for the next iteration of the model. For now we have clarified the scope of the model and mention lipids as a planned development in Discussion. Carbohydrates are less well studied in salmon, and we have limited our revision to clarifying that these are not represented in high resolution in the model.

* L163: Only at this point in the manuscript I understood the scope of the model (energy, amino acid, and nucleotide metabolism). I believe the abstract, introduction and any other summary section of the manuscript should clearly highlight this scope and mention that this is a smaller-scale / highly-curated model and not genome-scale model, otherwise it reads misleading, making the reader think that this model is part of the genome-scale family. Furthermore, the authors should provide a richer discussion about what are the advantages/disadvantages of generating such a model instead of using the (more prevalent) full genome-scale model approach.

The scope of the model was also brought up by the other two reviewers, and we have revised the whole text to make it clear that the model focuses on energy, amino acid, and nucleotide metabolism (see our reply to reviewer #1). We have done our best to justify bottom-up reconstruction in Discussion:

"Although laborious and time-consuming, our bottom-up manual reconstruction of the Atlantic salmon metabolic network was necessary to make SALARECON a high-quality predictive model. Automatically built models work well for microbes but are still outperformed by models that are built by manual iteration, and reconstruction of eukaryotes is more challenging due to larger genomes, less knowledge, and compartmentalization."

* The model repository is quite unorganized and could benefit from some cleanup: I would recommend following a standard (e.g. https://github.com/SysBioChalmers/Human-GEM or the broader https://github.com/drivendata/cookiecutter-data-science). Specifically, the following ideas would increase readability of the repo:

We thank the reviewer for these references, which were not familiar to us. The README of the repo was meant to serve as a concise guide for the reader, with the Jupyter notebooks providing

reproducible code for the methods and results in the manuscript. We have retained this, but completely reorganized the repository to meet the reviewer's suggestions.

* It is confusing to find the model within a plethora of genome-scale models from different species. Furthermore, is it even needed to store models from other studies in this repo? Providing the links from BiGG should be enough.

We have followed the reviewer's suggestion, and will push the restructured repo shortly.

* Having a folder with old versions of the model defeats the purpose of using git: Shouldn't those models be accessible through specific commits in the git history? Labels can be provided to those commits for quick reference.

While we understand the reviewer's argument, we feel there is tension between the principles of version control and the need for model comparison. It is a very common occurrence that one wants to compare a model to a previous version or to a model from a different repository. Checking out the entire repo to that revision would affect many files and also overwrite the current version to be compared. MEMOTE is a big, sluggish machine, whereas for many purposes we also rely on ad hoc programming of comparisons and visualizations.

As a compromise, we now treat old revisions for comparison the same as external model files: check if a cached copy of the version to be compared exists, otherwise programmatically copy it from the git history using git show. This is analogous to programmatically downloading external models from BiGG.

Minor comments

* L39: Please detail (in methods or in supplementary material) the settings that were chosen for WoLF PSORT and SAPP.

We used default settings for WoLF PSORT and SAPP was used an interface for running WoLF PSORT. This is now clarified as follows in the methods section:

"We used WoLF PSORT with default settings through SAPP to assign metabolites and reactions to six different compartments."

* L44: Unclear to me what "We iteratively converted the model to the BiGG namespace" means: why would the authors need to convert the model ids more than once to BiGG?

We agree that this was unclear. The only "iterative" process was adding or removing reactions; the conversion of identifiers is indeed a one-time operation. We have reworded this as follows: "After finishing the draft model, we converted the model to the BiGG namespace and used COBRApy to iteratively curate it."

* L56: It is unclear to me what was the exact criteria for stopping model annotation/curation.

*We have added a parenthesis to emphasize that the quality criteria are detailed below, and now state the scope of the model at the end of the sentence in question, lines 64-67 of the revised manuscript: "We finalized the model by alternating semi-automated annotation and curation with quality evaluation **(as decribed below)**, iterating until we saw no further opportunities to improve the model without expanding its scope **beyond energy, amino acid, and nucleotide metabolism**".*

* L80: For a randomized approach like the one the authors present, 100 randomized conditions is probably not enough for properly sampling the solution space. I would recommend the authors increasing this number to at least 1000. On the other hand, 100 linearly spaced oxygen uptake rates is probably redundant and could be replaced with a smaller number, considering that the response of the model seems to be more or less linear (Fig. 4d).

As detailed in our response to Reviewer #2, we have redone the sampling with 1000 realizations and improved our random sampling procedure, with very similar results.

* L89: Here I am still confused: as far as I understand there were 100 (feed random uptake combinations) * 100 (O2 linearly spaced O2 uptakes) = 10,000 simulations performed. Are there also other reaction bounds randomly sampled?

As stated in our response to Reviewer #1: Each condition is defined by randomly sampled ratios of nutrients and randomly sampled upper bounds for reactions. We have significantly revised the oxygen-limited growth analysis and expanded and clarified its description under Methods, which now includes the following explanations:

“We used parsimonious FBA (pFBA) to find maximal growth rates and minimal flux distributions for 1,000 randomized conditions and 50 logarithmically spaced oxygen uptake rates. (...) For each condition, we uniformly sampled random ratios (1-100) of nutrients in a minimal feed (essential amino acids and choline) that were used as coefficients in a boundary reaction representing feed uptake. (...) We did not allow uptake of any other compounds than essential amino acids, choline, phosphate, and oxygen under any condition. (...) To account for uncertainty in relative flux capacities and ensure that no single reaction was always growth-limiting, we also sampled random bounds for all reactions for each condition.”

* L133: If the authors claim that the node degree distributions of the network "are typical for metabolic and other biological networks", then I would expect Figure S1 to show numbers for those networks, however only the information for the salmon model is presented.

This claim is supported by the reference to Broido, A.D., Clauset, A. Scale-free networks are rare. Nat Commun 10, 1017 (2019). https://doi.org/10.1038/s41467-019-08746-5

* Figures 3a, 3d and 4c are not referenced anywhere in the manuscript as far as I can tell.

We thank the reviewer for pointing this out. Fig. 3a was cited in line 147, but fig. 3d should have been cited in line 164 after "SALARECON correctly captured all expected metabolic

(in)capabilities for the three metabolic systems within the scope of the model (energy, amino acid, and nucleotide metabolism; fig. 3c and 3d)." This has now been fixed.

* Fig. 3d: Amino acid essentiality data should be shown together with the predictions here, otherwise the statement in the caption "Essential amino acids predicted by SALARECON match data" is not proven.

The original only had one subfigure for this since predictions agreed with data. For clarity, Fig. 4d now shows the same subfigure twice under headings "Predicted" and "Observed" to explicitly show the agreement.

* L205: When performing pathway enrichment, it is standard practice to show in the plot (Fig. 4f) the number of reactions that each pathway contains, as a pathway with less than e.g. 5 reactions in the model should probably be filtered out, given that already a couple of reactions showing in a cluster could be counted as significantly over-represented. Note that the filtering here should be done by reactions, not genes (to not account for the same flux value multiple times).

The pathway enrichment was performed using genes rather than reactions because we did not have pathway classifications for the reactions. The minimal number of genes across all enriched pathways was 7.

* L263: "Automatically built models work well for microbes but are still outperformed by models that are built by manual iteration" This is a strong claim that I believe requires at least some references and explaining what is meant by "outperforming".

The claim was meant to refer to eukaryotes; we do not dispute that automatic reconstruction has come to be very successful in prokaryotes. In Discussion, we write "Although laborious and time-consuming, our bottom-up manual reconstruction of the Atlantic salmon metabolic network was necessary to make SALARECON a high-quality predictive model. Automatically built models work well for microbes but are still outperformed by models that are built by manual iteration, and reconstruction of eukaryotes is more challenging due to larger genomes, less knowledge, and compartmentalization."

* L277: Comparing the memote score of this model to models in BiGG is in my opinion unfair, as all those models were published before the memote score was introduced, and therefore did not account for it in their development. Furthermore, by adding a few things to those models, such as annotation and/or SBO terms, many of those models would increase their score to close to 95% as well.

While this is true, we believe the low MEMOTE scores of BiGG models reflect genuine room for improvement. We now mention the following in Results: "(...) although many BiGG models could presumably be annotated and curated to reach a comparable score with reasonable effort".

* L295: This is achieved only because the biomass reaction is relatively simple (e.g. one type of carbohydrate and one type of PC), so I'm not sure it should be highlighted as a great advantage of the model.

Point taken; we have reworded this to "a fundamental sanity check" and moved it earlier in the discussion.

* L320: Check grammar of sentence.

Thanks; this was missing an "of" before "the Atlantic salmon microbiota". Fixed now.

* L356: Should say S3 Fig.

Fixed; thanks.
