# Supplementary material for: SALARECON connects the Atlantic salmon genome to growth and feed efficiency
Source: PLoS Comput Biol. 2022 Jun 10;18(6):e1010194. doi: 10.1371/journal.pcbi.1010194 (PMC9223387; doi:10.1371/journal.pcbi.1010194)
Supplement: Attachment — Submitted filename: salarecon_revision_response_reviewers.pdf [file pcbi.1010194.s013.pdf]

Dear Editor,

Thank you very much for the invitation to revise our manuscript. We are very happy that reviewer #1 is now fully satisfied, and believe we have accommodated the insightful points raised by reviewer #2. A point-by-point response to their comments follows below.

Reviewer #2: For this revision of their paper on metabolic modeling of Atlantic salmon the authors have developed the description of their methodology and have expanded the biological analysis. However, some questions remain to be addressed as detailed in the following specific comments.

Major comments

1. The authors maintain that Jacard distance is indicative of similarity between fish metabolism and a metric of salmon-specificity, “The models clustered by phylogeny with fish and mammals forming distinct groups and the diatom as an outlier, indicating that SALARECON captures fish- and likely salmon-specific metabolism.”. If these models were all genome-scale reconstructions, this may be a correct conclusion, however, since many reactions are missing from SALARECON due to its limited scope, it is not apparent if the clustering is driven by similarity in scope or in content. As an illustration: if every reaction in the salmon reconstruction is also present in the human reconstruction, the Jaccard distance would still be very large ~0.85 (1 - 456/3554), due to difference in reconstruction size, while the corresponding value for zebra fish would be 0.65, and in such case they would thus cluster due to similarity in scope. At best the results are consistent with the hypothesis that SALARECON captures fish and salmon-specific metabolism, but Jaccard distance does simply not seem like an appropriate metric when comparing reconstructions of different scope.

That is a good point. For a more nuanced portrayal of the similarities and differences of metabolic models for the different organisms, we have redone the clustering using all the suitable dissimilarity measures and clustering algorithms available in the SciPy software, then computed the "cophenetic correlation coefficient" for each combination of measure and algorithm.

We describe in Results that "models tended to cluster by phylogeny with fish and, to some extent, mammals forming distinct clusters and the diatom being an outlier. [...] This is largely consistent with the hypothesis that the models capture organism-specific metabolism, suggesting that SALARECON captures salmon- or at least fish-specific metabolism. However, there are also significant discrepancies between trees built with different measures and methods, and it is important to note that the clusters likely reflect large differences in model scope as well as organism specificity. [...]"

We include a new supplementary Fig. S4 illustrating the variation in dendrograms using the different methods. For all revised text, see the highlighted portions of the first paragraph of "Evaluating the quality of the metabolic model" in Methods, the second paragraph of Results, and the caption of Fig. 3a.

We believe that our revised version gives a fair portrayal of the species comparisons as well as the inherent difficulties in assessing the extent to which clustering reflects biology or model scope, and thank the reviewer for their clear statement of the issue.

2. Similarly, in Figure 2e it is not clear if the observed differences are due to difference in species or reconstruction size, all differences seem to suggest that the Human model has additional capacity, e.g. for the human model, the phosphate likely originates from phospholipid metabolism, which is not reconstructed in SALARECON. It is not clear if SALARECON has any metabolites or reactions that are not also present in the human reconstruction, and it is not clear if the reactions or metabolites that are missing in SALARECON are due to limited reconstruction scope or genuinely missing.

Again a valid point. We have clarified the text by extending the paragraph discussing Fig. 3 (sic; Fig 2 has no panel e) with "In general, RECON3D captures a much larger space of possible growth-associated metabolic activities than SALARECON due to the large difference in model scope. However, SALARECON specifically captures the key metabolic activities of a fish."

3. The Monod model with an intercept term is not the Monod model. It is perhaps an “Extended Monod model”. However, I the r/r _max term was used instead of x in the Monod equation, there would likely be no need for an intercept and the results would be more comparable to the model as they receive the same input.

We have clarified the phrase to "a Monod model extended with an *x*-intercept" in Methods. Elsewhere in the text we now refer to this model as an “extended Monod model”.

4. Regarding the choice of uniform distribution for nutrient sampling, the Authors write in their point-by-point response “we do not have prior information and therefore choose to sample from a uniform distribution”, however, is not the fish meal in Fig.5a such prior knowledge?

The reviewer is correct that we do have knowledge about fish meal composition. We have therefore repeated the oxygen-limited growth analysis with a fish meal feed and default bounds, and we show the results in the new Figs. S9 and S10. Predictions and parameter estimates are not very different from those obtained with randomly sampled feeds and bounds, but sampling allows us to account for more uncertainty. Also, we do not have information about feed composition for the experimental data.

5. The capacity constraints are sampled from a log normal distribution, spanning 6 orders of magnitude. How do the authors ensure that the uptake constrains are of a corresponding magnitude? The manuscript states that uptake is normalized to 1 g/gdw/h, and that this value is arbitrary, however, it will not be arbitrary if the flux capacities are constrained, e.g. if uptake is much larger than capacity, then capacity will determine growth, and if uptake rates are much lower than capacity, then uptake will determine growth. To ensure that both constraints are active the authors could compare the curve in Figure 4a with and without uptake constraints. Alternatively, the predicted fluxes could be compared with the constraints to determine the frequency of constraining growth (flux==constraint) for each internal- and uptake reaction.

We deliberately chose the normalization of the feed uptake reaction such that feed uptake (which covers all uptake except oxygen and phosphate) would not be limiting for growth, i.e. such that internal flux capacities would always be limiting. We have now made this

clear in Methods as follows: “The absolute value was selected to be large enough to ensure feed uptake was not limiting but otherwise arbitrary as only relative predictions were needed”. Our new analysis of limiting reactions for oxygen-limited growth confirmed that feed uptake was not limiting in any of the predicted flux distributions (Fig. S10).

We do not have good prior knowledge of flux bounds nor which reactions should be expected to be limiting, but this is addressed by random sampling of flux bounds. As shown in Fig. S10, this sampling allows us to account for a much larger selection of potentially limiting reactions than we do with the default bounds. For this analysis, we have done exactly what was suggested by the reviewer, i.e. compared fluxes to bounds.

6. The revised sampling method resembles the one used by Beg et al 2007, further described by Adadi et al 2012, it may be worth checking if they are mathematically the same as the proposed method.

We thank the reviewer for the helpful references. The approaches used in these two papers (FBAwMC and MOMENT) account for molecular crowding and enzyme kinetics in FBA predictions and differ fundamentally from ours. As far as we can tell, these methods do not involve random sampling of feed/medium compositions or flux capacities.

7. The authors state that the purpose of figure 4 is to “show that SALARECON can give good estimates of physiological parameters (minimal and maximal water oxygen saturation)”. However, the minimal water oxygen saturation is a fitting parameter (x0) and the maximum is another fitting parameter (x1), so it is not clear that the results achieve this purpose.

We agree that Fig. 4 is a model-fitting exercise. Our point is that using SALARECON to fit to empirical data on oxygen availability ensures consistency with knowledge about the metabolic network in a way that is out of scope of the simpler models. We do believe that the reviewer’s statement is entirely compatible with our Discussion sentence “phenotypes predicted by SALARECON can be fit to experimental data and produce detailed mechanistic explanations of Atlantic salmon physiology”.

Minor comments:

1. The definition of Jaccard distance is in equation 1 seems to be the Jaccard similarity, for distance: 1 – J(A, B).

This is entirely correct, and we apologize for the mixup. It has been corrected as part of the rewrite in light of major comment 1.

2. It may be contested that Robinson et al. 2020 have reconstructed a more recent human model than Recon3D, but perhaps this is the most recent model in the BIGG database.

We have rephrased our reference to Recon3D as "one of the latest human models".

3. The authors sample the upper- and lower bounds of reversible fluxes independently. Since the [E] term is present in both the forward and backward reaction, they are in principle not independent. However, in practice this will not matter since pFBA ensures that each reaction will be either in the forward or backward direction.

We agree with the reviewer's point and the conclusion that our results are not affected.

4. Figure 3c says "fitted value" on y axis, but R2 is the calculated coefficient of determination, not a fitted value.

We have removed the words "parameter" and "fitted" from the axis labels.

5. Figure S6F shows absolute growth rate and uptake rates. The growth rate is 1% per hour, which is approximately 24 times higher than observed in salmon Cook et al 2000.

As noted previously, the absolute values of these growth rates are not meaningful, nor were they meant to be. We have added this to the legend of Fig. S7 (previously Fig. S6): "The absolute growth rates were not intended to be realistic and only relative growth rates were used in the analysis (normalized by maximum growth rate without oxygen limitation)."

6. In figure 2e the figure legend could perhaps be more clearly state that slash ("/") indicates sets of metabolites, e.g. NH3 is present in both the red and blue box, but this is presumably because it is compared against the whole set NH3/Urea/Urate.

We have clarified the legend to Fig. 3e (sic; Fig. 2 has no panel e) to state "Ammonia secretion can be replaced by urea or urate secretion in human but not in salmon". We also added the word "only" to the sentence "Urea is the major nitrogenous waste in mammals, but RECON3D could grow while secreting only ammonia or urate" in the main text.

References

Beg, Q. K. et al. Intracellular crowding defines the mode and sequence of substrate uptake by Escherichia coli and constrains its metabolic activity. Proc. Natl. Acad. Sci. U. S. A. 104, 12663–12668 (2007).

Adadi, R., Volkmer, B., Milo, R., Heinemann, M. & Shlomi, T. Prediction of Microbial Growth Rate versus Biomass Yield by a Metabolic Network with Kinetic Parameters. PLoS Comput. Biol. 8, e1002575 (2012).

Robinson, J et al. An atlas of human metabolism. Sci. Signal. 13, eaaz1482 (2020).

Adadi, R., Volkmer, B., Milo, R., Heinemann, M. & Shlomi, T. Prediction of Microbial Growth Rate versus Biomass Yield by a Metabolic Network with Kinetic Parameters. PLoS Comput. Biol. 8, e1002575 (2012).

Cook, J. T., McNiven, M. A., Richardson, G. F. & Sutterlin, A. M. Growth rate, body composition and feed digestibility/conversion of growth-enhanced transgenic Atlantic salmon (Salmo salar). Aquaculture 188, 15–32 (2000).
